# The Jenkins System of Porcelain Fillings

**Published:** 1899-02

**Authors:** R. Ottolengui

**Affiliations:** New York.


ARTICLE II.

### THE JENKINS SYSTEM OF PORCELAIN FILLINGS.

R. OTTOLENGUI, M. D. S., NEW YORK.

**Read before the Central Dental Association of Northern New Jersey, October. 1898.**

On the day when I began the study of dentistry my preceptor put me out in the laboratory and gave me a book to read which I think was Harris's Principles and Practice of Dentistry; I opened the book at haphazard and read somewhere the constituents of enamel, and I immediately concluded that I would be the one to invent the ideal filling; that I would find the means of taking the constituents of enamel and putting them together so that they might be used in a tooth as a filling. That was some twenty-one years ago, and I have not yet found the formula for mechanically producing enamel. Yet tonight I am presenting, and to some extent advocating, a system which perhaps is the highest achievement in this direction.

It is hardly necessary for me to go over the history of what may be called porcelain filling, except briefly. We all remember the porcelain fillings which were made by Land, and which were not all that was claimed for them; then the glass filling of Herbst and later on a material which was furnished by dental manufacturers and largely used by dentists and other men in Europe, which was called porcelain, but which was really glass. Those fillings I think might be considered a high fusing glass; it was a glass which could be fused on gold foil without melting the gold. This seemed to be an advance over the Land method. So far as the mechanical difficulties in the way were concerned, I think they were met fully by this German preparation, but it was not durable and cracked readily in the mouth, even in places where no strain (or I believe the Western word is "stress") was put upon it. Dr. Jenkins imagined that he could improve on this, and did to some extent by flowing glass over the filling after it was made, thus accomplishing two things which were eminently satisfactory; first, the filling could be put into the mouth and ground and then this glass could be flowed over it, the glass fusing at a lower degree than the body of the filling. Second, a lustrous surface was thus produced. But even with this Dr. Jenkins soon discovered that he had not yet reached an ideal method of work.

I believe I am rightly informed when I state that during his various holiday seasons he has visited nearly all of the glass and porcelain factories in Germany, and it was as a result of these many visits and his study of the various bodies used in the works of that region that he has eventually produced the porcelain which he is now introducing. I say introducing, because Dr. Jenkins writes that he has not yet surmounted all the difficulties in the way of manufacture; that even to produce the eighteen shades of porcelain which he furnishes, so that each mass or body shall always have the same shade is exceedingly difficult, and it is also a very expensive process. But he believes that some of the difficulties are gradually disappearing under the hands of those who are making the porcelain under his guidance, and that in the future it may be possible to produce the material in an even more satisfactory form and at a more satisfactory price.

What is the ideal filling? It seems to me that the ideal filling, as in everything else, must depend somewhat upon the mind which conceives the ideal. The ideal of one young women is a blond man and the ideal of another young women is a brunette, and it would be very difficult to say, speaking generally, whether the blond or the brunette is really the ideal man. So it is with these fillings; Dr. Jenkins has reached the absolute ideal for Europe, but I question very seriously whether he has reached the ideal for America. The American people are becoming national; originating from hetergeneous sources they are becoming rapidly homogeneous in national character, and if there is one predominating characteristic in the American, it is that utility, practical utility, must supersede everything else; estheticism or any artistic standard is secondary in the mind of the American to utility. Thus we find that in Europe the people object to a display in the mouth of anything that touches on artificiality. The American desires that his artificial teeth may seem not to be artificial; so much so that many dentists to-day say, " we hope that we do not make *false* teeth; we make *artificial* teeth." But Americans have not been educated up to the point of considering that there is any objection to having a part of the tooth false. Europeans do. I cannot refrain from repeating a little incident which I have told before: A European noble lady once came into my hands for a very simple service; she wished a porcelain crown reset. I pointed out to her that several of her incisors required filling, and she said " Yes, but I must return to Europe before I can have them filled." That rather surprised me, who had been taught that American dentistry surpassed all other dentistry in the world, and I asked her "Why?" and she replied, "Because I cannot get anything in this country but gold; in America you fill teeth with gold, and in Europe we fill teeth with something the color of the teeth." I answered "Yes; but we can fill a tooth with that white material, if you wish it, in this country." She said, "Can you? Then why do you not do it?" and I replied, "Simply because in this country we prefer what is permanent, and those white fillings are not permanent." "Oh," she said, "that is just my objection; I would rather be temporarily beautiful then permanently hideous!"

That voices the position of practically 99 per cent of Europeans; they would rather have something temporarily in their teeth which does not show, than to have the permanent gold fillings of the Americans. Undoubtedly there are some Americans who would feel the same way, but how many of them are there? In America the question is, "Will it last?" and patients will come in and complain that a filling which was put in *only five years ago*, is out. So the standard of the ideal is different. Europeans have been accustomed for the last twenty-five years, when they have been unfortunate enough to have cavities in the front of their mouths, to return to the dentist once in a year or in two years to have "those nice white fillings" put back. Consequently the porcelain filling, which looks so much more natural, is the ideal filling for Europe, even if it must be replaced every two or three years, although they will probably, in many instances, last much longer. But in America whilst, as I have said, there are some people who feel that they prefer to have the porcelain filling because it does not show its falseness, I believe that the great majority of them, if you introduce the subject of porcelain fillings, will say, "Will it last as well as gold?" and that will be a serious question for the *honest* dentist to answer; because the standard to-day in this country, I am proud to say, is higher in the matter of filling teeth, than it ever was. The requisites to-day for making a perfect filling are more rigid and more exacting than ever. The man who puts a filling in to-day puts it in to stay not for a day, or a month, or a year, but forever, and ther are thousands of dentists in this country who can make gold fillings which will endure and not have decay recur around the margins. If this question of the margin of gold fillings has become so important that the dentist of to-day before filling a tooth cavity polishes the edge and examines it again with a magnifying glass of ten or twenty diameters, you cannot attain that standard which we would call ideal, even if you do match the color, unless the margins of the porcelain fillings can be made as absolutely perfect as the gold fillings; and while I shall in a moment advocate the Jenkins method, I wish it to be distinctly understood that I limit it in its application to those places where the margins can be made as perfect as they are with gold, and I ask every dentist who hears me and who undertakes this work to feel dissatisfied with any porcelain filling until the margins are as perfect at every point as he could expect them to be with gold filling.

To a limited extent the Jenkins system attains that requisite; that is to say, where the cavity is accessible and care and skill are used; a porcelain filling with the Jenkins body can be made, which, when placed in the cavity will have such perfect margins that the eye of man cannot detect the point of union. At that moment we come to a difficulty, and that is the cement. These porcelain fillings by the Jenkins method are made with No. 30 rolled gold, and of course that means that there is a very slight space between the filling and the bottom of the cavity, and there is no cement in the market in America known to me, which can be mixed so thin as to occupy that space which was occupied by the gold filling, and occupy no more space, and have strength and adhesiveness to retain that filling in the cavity. A curious thing has been told me by manufacturers of phosphate cement, and that is that climate has such an effect upon the cement that those cements which are vaunted and relied upon for this work in Europe are worthless in this country, because of our climate, and *vice*

*versa*, the cements which are very excellent for filling purposes and for attaching crowns in this country have proven failures on the other side, and have been returned with the request that the money be refunded. I do not to-day know a phosphate cement that I can obtain and use in this country with which I can set a porcelain filling to my entire satisfaction. By that I mean that I would like to be able to set a porcelain filling so that when set *absolutely no cement is visible.* In other words, a cement which would be so thin that it would occupy no more space than the matrix did. I have used a number of cements for this work and have discarded them. About a year or more ago I used the German Fused Oxide, but discarded it; since then I have discovered that there is another form of it, the powder of which is so fine that I can make a very smooth cement, whereas the lot I had at first was very gritty and consequently absolutely unfit for the purpose of cementing these inlays. With the German Fused Oxide I am getting better results than with anything else that I have yet tried, but I say that before we can have the ideal in this country, a cement must be made so as to be used very thin, and I offer a suggestion to the men who are manufacturing cement; formerly we spoke of oxychloride *filling* and oxyphosphate *filling* material; now these are all spoken of as *cements*, but as far as I can judge the very same materials which were formerly called *fillings* are now called *cement*, simply because it happens that they have an adhesive quality. Now the principal requisite of a cement is totally different from the requirements for a filling material, and it is time for some manufacturer to give us two preparations, one of which shall be for *filling teeth* and the other for *cementing crowns, bridges and porcelain fillings* into position. Just think what a glorious thing it would be if we could have a cement with which we could put these fillings into position, which would be a liquid and which would have no powder in it! Is it impossible? What of Stratina; we see a plate broken in two and put together again with Stratina or some other of the liquid cements and no ordinany force will break that apart. Those materials cannot be used in the mouth because they take from twenty-four to thirty-six hours to harden, to set, and because they are not waterproof. But it ought to be within the limits of chemistry to give us a liquid cement, with little or no powder in it, for the cementing of these porcelain fillings, and when we get that we can indeed perhaps claim to have the ideal filling.

So much by way of comment, and I say as much as this because, curiously enough, a little article which Dr. Kingsley wrote in the Items of Interest has brought to him I suppose, two or three hundred letters from different parts of the country, asking for information on these various points. It has brought to me about three hundred letters, and I believe it has brought to the publishers of the magazine over a thousand letters, all of which indicates the interest that is taken throughout the country in the possibilities of this system.

There is perhaps nothing very new in the Jenkins system. The principal thing is that Dr. Jenkins has perfected many of the details which are familiar to all in connection with the old glass fillings; the chief advance being in the body itself.

The procedure is to take what might be called an impression of the cavity with rolled gold No. 30. That sounds very simple, but it is not only the most difficult part of the entire operation but I might say it is the only difficult part and the part upon which the final success depends. If the matrix does not give a perfect *replica* of the edges of the cavity, it will fail. The cervical margins which are always so essential in a gold filling are equally essential in a porcelain filling, and they are more difficult, for the reason that it is desirable to have your matrix extend over the surface of the tooth, that is, not to end at the cavity margin, but to extend over and lap down upon the surface of the tooth. Naturally the nearer you get to the gum the more difficult it is to deal with that portion of the margin. The matrix must extend beyond the cavity, but it extends now at an oblique angle, or almost you might say, vertically, and unless the impression of the edge is exceedingly sharp you will run the risk of building the body higher at that point than it should be. This must be remembered, that in order to get the matrix perfect, the cavity must be so shaped that the gold may be removed without changing its form, and in order to accomplish that it is necessary in dealing with many cavities to cut away quite liberally If after cutting away all of the margins of the cavity that you feel warranted in doing, you should find that there are undercuts caused by the inroads of caries, it then becomes necessary to fill the tooth with phosphate cement; you cannot save time by simply filling the undercuts; the better way is to fill the whole tooth with phosphate cement and allow it to harden thoroughly and then to cut a cavity in which you can fashion a matrix which can be removed without alteration of shape. Subsequently of course when you come to set the filling this phosphate cement would be removed, and the undercuts serve to hold the new phosphate which retains the filling.

The cavity being prepared the essential feature is the treatment of the margins. The margins should not only be smooth, but they should be, if I may use such an expression, definite. I believe Dr. Jenkins advocates finishing the margins with diamond points, but I think yon can do well enough with very small and sharp gold finishing burrs. Where you can reach the margin with fine pouncing paper disks, I should advocate that; at any rate the margins should be smooth and rounded. By rounded I do not mean so beveled that there would be a thin edge of porcelain extending over it, but the sharp edge should be taken off, so that after fitting the matrix you can burnish it down against the edge without danger of cutting it through.

The next step is to form the matrix. This is done by taking a piece of foil of proper size, usually cut in an elliptical form, the corners removed. It is put lightly over the cavity and it is forced into the cavity lightly with spunk. It is then taken out and any extreme excess of gold is cut away. It is then replaced in the cavity and a small piece of spunk is placed in it, and another and another, the procedure being the same as when filling a tooth with soft gold; the cavity is simply packed tight with this spunk. The overlap of the gold matrix which may be standing up is wiped into position; that is to say the spunk which is in the cavity is held firmly down with a burnisher and another piece of spunk is taken in the tweezers and laid flat upon the external surface of the tooth by a wiping motion, so that when all the spunk is taken out the gold should lie smoothly in the cavity; perfectly along the cavity edges and smoothly on the outer surface wherever it overlaps. It has been advocated by some to smear the cavity with some emollient like vaseline; I have not done that nor found it necessary, because I think if you decide to fill with porcelain you might just as well cut away enough of the tooth to be able to get the matrix out, and if you do not feel warranted in doing that, you should fill the tooth with gold.

The matrix having been formed, Dr. Jenkins, mindful of the slightest details, furnishes us with a little celluloid box with a transparent celluloid cover; that is held right under the tooth and this matrix is what is called "teazed out," that is, it is tapped lightly until it dislodges itself; I say dislodges itself rather than that it is dislodged, but there is no better word for it than that it should be "teazed" out, and as soon as it is perfectly loose in the cavity it is gently tipped out and dropped into that box; the box is then covered and that matrix in that box will not change its shape. The patient may be sent away with a temporary filling and your porcelain prepared at your leisure.

When the time arrives to bake the porcelain a small platinum muffle is filled with powdered asbestos mixed with water to a paste. The upper surface of it sbould be fairly thin, milky; as some one suggested this afternoon, "condensed-milky." It should be thin enough so that the matrix when dropped in will sink down into the mixture. This is then placed in the furnace; the flame is then turned on and the heat is kept up until all the moisture has been driven out. Then we have the gold lying in the investment in exactly the same shape as it lay in the tooth and in a position where the shape cannot be altered.

Having chosen the appropriate color of body the powder is placed on a little slab, which is furnished. We are also furnished with a drop tube and a bottle labeled "Absolute Alcohol;" the powder of the body is mixed with absolute alcohol, and enough of it is placed in the matrix to fairly cover the bottom and sides. The muffle is then covered with a little cover which has a hole in it so that you can see in. This is placed in the furnace in a similiar manner as before, and the heat is brought up gradually until all the fumes of the alcohol have been driven off. During the operation a blue flame will be observed coming out of the muffle; that is the alcohol burning off. As soon as the blue flame stops you are safe enough to gradually draw the muffle over the hole until it rests over the center of the flame; the heat is then made more and more intense until the body fuses. The first fusing of the body will lead you to believe that there is very much shrinkage. It is taken out and cools off very quickly, because it is such a small affair, and as soon as it is sufficiently cooled to add more body, that is done. In adding the second portion of body care should be observed to get it down upon the gold, that is, wherever the shrinkage has caused the body to draw away from the matrix the new body should be mixed thin enough to pass under it and go down against the bottom of the matrix. The second fusing should fill up to the matrix so that you will feel satisfied that the body is in contact with the matrix throughout, and at the third baking you can usually build up your filling to the shape desired. This last fusing will present very little if any shrinkage. There is no particular necessity for care in fusing, excepting the last one; that is, it is the final body which gives you the color; even if you have overheated some of the first lot, that will not alter the color of the last lot, which will cover it over and give you the desired color. In doing this work, whilst arranging the various preparations of the body in the matrix, care should always be taken not to allow any of it to over-run the margins, because of course it will bake, and make thin overlays of enamel, which must be broken off afterwards, and it will also deceive you as to the height to which the porcelain should be built.

Having baked the filling, the gold is peeled off the back. It is not always that the gold will peel off very readily; if the bottom of your cavity is rough the gold will be correspondingly more difficult to remove, but I have found a very easy method of removing the gold when it does adhere. I use a sharp small burr in the engine and run it lightly over the back of the filling, the gold coming away nicely. The next step is to take all the gloss off the under side; that must be done even with the thinnest kind of inlay. Where there is sufficient thickness to permit it, undercuts should be made so as to give as rough a surface as possible to permit a proper adhesion of the phosphate cement.

All being in readiness to set the filling, my method has been to mix the cement as thin as I dared to make it and have stability to the cement; that is placed in the cavity and the filling is put into position and tapped into place quickly, the excess of phosphate oozing out. If this causes the filling to bulge from the cavity it should be removed immediately, and it should either be done over or more space for phosphate should be made, either by grinding the porcelain or by cutting away from the bottom of the cavity. When there is sufficient space for the phosphate to remain in the cavity, the filling is put in and tapped to place so that the cement oozes out all around it. If there is any reason to think the filling might become disturbed before the cement is thoroughly hardened, it should be tied in with a very narrow tape.

It may sometimes occur that the cavity may be so extensive that one would feel a hesitancy in putting in one of these fillings and expecting it to remain there unless something more than undercuts could be relied upon. In such cases I have put fillings in with pins in them. The method of putting in the pin is to produce a matrix in the way I have indicated, and after having fixed in your mind where it would be safe to put a pin in the tooth, the matrix is invested in the muffle and dried in the furnace, and then a very sharp pointed instrument is used to punch through the matrix at the point where you desire to put a pin; the investing material will not interfere with this, as it can readily be punctured. I then use a pin such as is used in ordinary porcelain teeth, pins in fact that I have taken from porcelain teeth. I crack up old teeth, take out the pins and always have a supply of them on hand. Take one of those pins, cut one end off and drop the other down so that the head alone rests upon the matrix, or if there is enough body let a little more than the head extend up; put one or two pins in, in this manner, and fuse the body around it in the usual way. Then you will have a filling n which there are two pins, and appropriate grooves must be cut in the cavity for the reception of these pins.

Now here is a valuable point about this body. It would be a very fortuitous circumstance to find that the pins were so placed as to be *exactly* where desired, and at *exactly* the proper angle to go into such a groove; you may determine in advance to cut a groove in a certain place and find that place so sensitive that you would prefer to have it elsewhere; this body is sufficiently dense to allow the pins to be bent in any direction without danger of fracturing the body.

In closing I would say that this work requires such skill, and such painstaking care to reach that standpoint which I consider represents the ideal of an American dentist, that unless you can be well paid for it, you cannot afford to do it.—*Items of Interest.*

